# A safe path to bariatric surgery: Mental health disorders in pre-operative patients

**DOI:** 10.1016/j.cpnec.2025.100300

**Published:** 2025-05-15

**Authors:** Andrew El Alam, Mohamad Fleifel, Hicham Baba, Souha Bayda, Bertha Maria Nassani, Jocelyne Azar, Arnaud Monier

**Affiliations:** a Endocrinology and Metabolism Division, Lebanese American University, Beirut, Lebanon; b Endocrinology and Metabolism Division, American University of Beirut Medical Center, Beirut, Lebanon; c Psychiatry Department, Lebanese American University, Beirut, Lebanon; d Internal Medicine and Clinical Immunology Division, Hotel Dieu De France, Beirut, Lebanon; e Endocrinology and Diabetology Division, Centre Hospitalier de Chartres, Louis Pasteur Hospital, Chartres, France

## Abstract

**Background:**

Obesity is one of the most significant global health concerns. As per the World Health Organization (WHO), obesity currently affects nearly 1.9 billion individuals around the world. Patients suffering from such a metabolic disease exhibit multiple medical conjoint medical conditions, and are predisposed to future high-morbidity and mortality complications. In addition, such patients might suffer from psychiatric compromises, at any time during their lives, that might have contributed to obesity. For many of these patients, bariatric surgery remains one of the leading methodologies in facilitating weight loss.

**Aim:**

To study the prevalence of selected mental health disorders history, including depression, childhood trauma, and eating disorders, plus abnormal eating behaviors in patients with obesity undergoing pre-bariatric surgery evaluation. In addition, we intended to find any inter-associations between different mental health disorders and demographics in such patients.

**Methods:**

In this cross-sectional study, conducted at the Nutrition and Obesity Department at Louis Pasteur Hospital, France, we enrolled 234 patients with obesity undertaking pre-bariatric surgery evaluation.

**Results:**

Around 31.2 % of participants had a history of depression, with 46.5 % receiving treatment. Childhood trauma was identified in 22.6 % of patients, and 12.8 % exhibited eating disorders, subclassified into binge eating disorder (6.4 %), bulimia (3.2 %), and night eating syndrome (3.4 %). Abnormal eating behaviors was also prominent in such patients, with 66.2 % engaging in activities such as snacking, hyperphagia, emotional eating, and compulsive eating. Sociodemographic associations showed that females were more likely to be diagnosed with depression, binge eating disorder, and compulsive eating, while males were more prone to hyperphagia. Childhood trauma was significantly associated with depression, binge eating disorder, bulimia, and abnormal eating behaviors. Multinomial logistic regression analysis revealed various predictors for depression, eating disorders, and abnormal eating behaviors across different categories. Notably, depression was associated with unemployment, trauma, and compulsions. Binge eating disorder showed significant associations with trauma and the female sex, while bulimia was notably associated with trauma. Night eating syndrome was inversely related to marriage status. Subgroup analysis further highlighted associations between depression, eating disorders, and abnormal eating behaviors in specific demographic groups.

**Conclusion:**

There is a complex link between mental health disorders and eating patterns in individuals with obesity undergoing pre-bariatric surgery evaluation. Understanding this association is important for developing comprehensive preoperative care strategies that address both physical and mental health aspects in the management of obesity.

## Highlights

• There is an importance of addressing mental health concerns in the pre-surgery phase.
• Depression was identified in pre-bariatric surgery patients, revealing a potential link between early life experiences and adult obesity.
• Childhood traumas were highlighted in pre-bariatric surgery patients.
• Many participants showed abnormal eating behaviors, emphasizing the complexity of eating patterns in the context of obesity.
• There was a relationship between depression in participants with obesity and socioeconomic factors such as unemployment.

## 1 Introduction

Obesity is a metabolic pandemic that affects nearly 1.9 billion individuals worldwide as per the World Health Organization (WHO), which comprises approximately 39 % of the overall population [1,2]. This chronic condition is influenced by various factors, including genetics, environment, socioeconomics states, metabolic conditions, and mental health [1]. Individuals suffering from obesity face an increased risk of various medical morbidities and future mortality if not well addressed. They are also susceptible to social stigmatization and a range of psychological challenges like depression and eating disorders, which the latter includes binge eating disorder (BED), night eating syndrome (NES), and bulimia nervosa (BN) [3,4]. One of the contributing factors to eating disorders, in general, is childhood trauma [5] (see Fig. 1).

**Graph 1:**
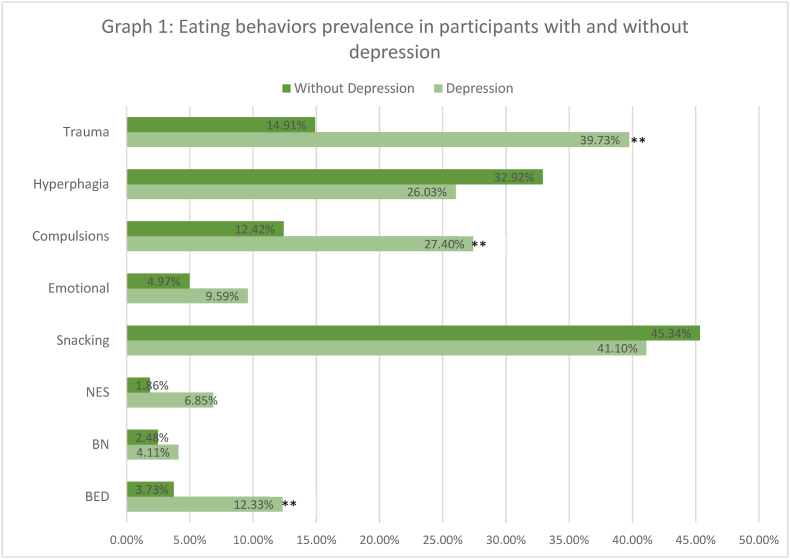
Eating behaviors prevalence in participants with and without depression.

When evaluating findings from France, the 2020 edition of the Obepi-Roche study showed that around 17 % of the French population is regarded as having obesity (BMI ≥30 kg/m2), with approximately 2 % suffering from morbid obesity (BMI ≥40 kg/m2) [6]. It appears that adults with obesity (aged 18 and above) suffering from depression show a significant dynamic bidirectional relationship in accordance to French geographic distribution, with a high prevalence of obesity being displayed in regions with prominent depression, and more likelihood of finding a high prevalence of depression in regions with high obesity [7]. A cross-sectional study from Bordeaux, France, which surveyed 1142 individuals while assessing anxiety/depression (Hospital Anxiety and Depression Scale), emotional eating (Emotional Appetite Questionnaire), food addiction (modified Yale Food Addiction Scale), and intuitive eating (Intuitive Eating Scale-2), found that individuals with obesity reported higher depression and emotional eating levels than other weight classes participants. They also reported experiencing more severe and prominent food addiction symptoms. When investigating associations, all weight classes exhibited depression, anxiety, food addiction symptoms, and difficulties to rely on hunger and satiety [8].

Currently, one of the treatments for obesity is bariatric surgery which has been revolutionary significant in long-lasting weight reduction. It is acknowledged that the success of bariatric surgery hinges not only on achieving weight loss, but also on the enhancement of mental health status [2]. The aim of this cross-sectional study was to determine the prevalence of selected mental health disorders, which include depression, childhood trauma, and eating disorders plus abnormal eating behaviors in patients with obesity undergoing pre-bariatric surgery evaluation. In addition, we intended to find any inter-associations between different mental health disorders and demographics in such patients. According to our research, there is no current study, in the English literature, that evaluates this topic.

## 2 Methods

### 2.1 Study design and sample

This is a cross-sectional observational retrospective study that was conducted at the Nutrition and Obesity Department at Louis Pasteur Hospital, France. The study sample consisted of 248 patients with obesity, 18 years old and above, who were referred for pre-bariatric surgery evaluation.

The inclusion criteria were patients above 18 years with BMI>30 kg/m2 referred for pre-bariatric evaluation. Patients who have already undergone a bariatric surgery, those younger than 18 years, and for whom there were language barriers preventing a fully accurate medical and psychiatric evaluation, were excluded from the study.

### 2.2 Data collection

Consent was obtained from each patient to access their records, which was also granted by the Hospital. All information was collected and anonymized in compliance with privacy laws to ensure anonymity.

Psychiatric information was derived from evaluations conducted during the preoperative phase by psychologists or psychiatrists using a structured clinical interview to assess for comorbidities, emotional well-being and presence of eating disorders.

Demographic, physiological, and psychiatric variables were collected. These included the patient's age, sex, BMI, hemoglobin A1c (HbA1C) level, employment status, and marital status. Psychiatric data included screening for depression via the Patient Health Questionnaire-2 (PHQ-2) [9], ongoing depression treatments, trauma history, and diagnosed eating disorders, categorized into BED, BN, and NES, according to the Eating Disorder Examination Questionnaire (EDE-Q) [10] and Night Eating Questionnaire (NEQ) [11]. Results of the scales were calculated and charted qualitatively accordingly.

Furthermore, we recorded instances of abnormal eating behaviors that did not fall under any formal diagnosis, such as snacking, hyperphagia, emotional eating, and compulsory intake. The process of data extraction and entry was performed systematically to maintain consistency and reduce potential bias. The study did not include any individual with missing data; the potential bias and distortion that can result from inputted missing values were removed by using the listwise deletion method, which contributed to maintaining the accuracy and integrity of the statistical analyses.

### 2.3 Data analysis

We performed descriptive statistics. Categorical variables were expressed as absolute and relative (%) frequencies. Age has been divided into five categories (<35, 35–44, 45–54, 55–64, and ≥65) due to potentially differing long-term outcomes [12]. Obesity classes were divided by WHO classification (class I < 30–35>, class II severe <35–40>, class III very severe - morbid >40). Individuals with HbA1C > 6.5 % all had type 2 diabetes mellitus (T2DM), whereas <6.5 % was reserved for non-T2DM, preT2DM, or controlled T2DM.

Chi-square test and the Fisher exact test were used for the analysis of categorical variables. Additionally, multinomial logistical regression with backward elimination was performed to understand the relationships between independent variables and dependent variables. For all statistical tests, a significance level of α = 0.05 was considered. P-values below this threshold were deemed statistically significant. SAS JMP Pro 17.1.0 software was used for all calculations and analysis.

## 3 Results

### 3.1 Descriptive

248 patients were identified for this study, 14 of whom had missing HbA1C levels. Thus, data was analyzed for 234 patients. Of these, we determined 54 % females (n = 126) and 46 % males (n = 108), with a majority being married (69.7 %, n = 163) and working (65 %, n = 154). Ages ranged from 18 to 81 years old (median = 44, mean = 44), and BMI ranged from 30 to 80 kg/m2 (median = 42, mean = 42.6). 21 % had T2DM (n = 50). There were no significant differences between males and females in marriage, age, BMI, and T2DM status, whereas males were more likely to be working (Chi-Square 4.8, p-value 0.029).

#### 3.1.1 Rates of depression, childhood trauma, and eating disorders

Depression was present in 31.2 % of the participants (n = 73), with 46.5 % (n = 34) of these individuals having on-going treatment. Childhood trauma was identified in 22.6 % (n = 53) of patients. Eating disorders were prevalent in 12.8 % (n = 30) of the population. More specifically, BED was diagnosed in 6.4 % (n = 15), BN in 3.2 % (n = 8), and NES in 3.4 % (n = 8). Abnormal eating behavior was seen in 66.2 % (n = 155), with 41.5 % (n = 103) involved in snacking, 29.0 % (n = 72) in hyperphagia, 6.0 % (n = 15) in emotional eating, and 16.1 % (n = 40) in compulsive eating.

#### 3.1.2 Sociodemographic associations

Table 1, Table 2 show the breakdown of depression, trauma, eating disorders and behaviors along with sociodemographic categories.

**Table 1:** Distribution of depression, trauma, eating disorder and eating behaviors by sex, age, BMI, marriage, and diabetic status. Number per group is noted as absolute number, column% and row%. ∗∗ indicated significant finding with p-value <0.05.

	Depression (73) #N (column%, row%)	Trauma (53) N (column%, row%)	Eating Disorder (30) N (column%, row%)	Eating Behavior (155) N (column%, row%)
**Sex**				
Male	23 (31.5 %, 21.3 %)	14 (26.4 %, 13.0 %)	5 (16.7 %, 4.6 %)	80 (51.6 %, 74.1 %)
Female	50 (68.5 %, 39.7 %)∗∗	39 (73.6 %, 31.0 %)∗∗	25 (83.3 %, 19.8 %)∗∗	75 (48.4 %, 59.5 %)
**Age**				
<35	16 (21.9 %, 28.6 %)	11 (20.8 %, 19.6 %)	8 (26.7 %, 14.3 %)	37 (23.9 %, 66.1 %)
35–44	21 (28.8 %, 32.8 %)	15 (28.3 %, 23.4 %)	11 (36.7 %, 17.2 %)	45 (29.0 %, 70.3 %)
45–54	21 (28.8 %, 35.6 %)	17 (32.1 %, 28.8 %)	9 (30.0 %, 15.3 %)	40 (25.8 %, 67.8 %)
55–64	11 (15.1 %, 28.2 %)	7 (13.2 %, 18.0 %)	2 (6.7 %, 5.1 %)	24 (15.5 %, 61.5 %)
≥65	4 (5.5 %, 25.0 %)	3 (5.7 %, 18.8 %)	0 (0.0 %, 0.0 %)	9 (5.8 %, 56.3 %)
**BMI WHO Obesity Class**				
Class I	7 (9.6 %, 36.8 %)	3 (5.7 %, 15.8 %)	1 (3.3 %, 5.3 %)	16 (10.3 %, 84.2 %)
Class II	21 (28.8 %, 34.4 %)	18 (34.0 %, 29.5 %)	8 (26.7 %, 13.1 %)	35 (22.6 %, 57.4 %)
Class III	45 (61.6 %, 29.2 %)	32 (60.4 %, 20.8 %)	21 (70.0 %, 13.6 %)	104 (67.1 %, 67.5 %)
**Marriage Status**				
Married	52 (71.2 %, 31.9 %)	28 (52.8 %, 17.2 %)	17 (56.7 %, 10.4 %)	112 (72.3 %, 68.7 %)
Not Married	21 (28.8 %, 29.6 %)	25 (47.2 %, 35.2 %)	13 (43.3 %, 18.3 %)	43 (27.7 %, 60.6 %)
**Working Status**				
Working	37 (50.7 %, 24.0 %)	25 (47.2 %, 16.2 %)	18 (60.0 %, 11.7 %)	106 (68.4 %, 68.8 %)
Not Working	36 (49.3 %, 45.0 %)	28 (52.8 %, 35.0 %)	12 (40.0 %, 15.0 %)	49 (31.6 %, 61.3 %)
**Diabetes Status**				
Diabetic	14 (19.2 %, 28.0 %)	7 (13.2 %, 14.0 %)	5 (16.7 %, 10.0 %)	31 (20.0 %, 62.0 %)
Not Diabetic	59 (80.8 %, 32.1 %)	46 (86.8 %, 25.0 %)	25 (83.3 %, 13.6 %)	124 (80.0 %, 67.4 %)

**Table 2:** Distribution of BED, bulimia, NES, snacking, hyperphagia, emotional and compulsory eating behaviors by sex, age, BMI, marriage, and diabetic status. Number per group is noted as absolute number, column% and row%. ∗∗ indicated significant finding with p-value <0.05.

	BED (15) #N (column%, row%)	BN (7) #N (column%, row%)	NES (8) #N (column%, row%)	Snacking (103) #N (column%, row%)	Hyperphagia (72) #N (column%, row%)	Emotional (15) #N (column%, row%)	Compulsions (40) #N (column%, row%)
**Sex**							
Male	2 (13.3 %, 1.9 %)	1 (14.3 %, 0.9 %)	2 (25 %, 1.9 %)	53 (51.5 %, 49.1 %)	43 (59.7 %, 39.8 %)∗∗	3 (20 %, 2.8 %)	12 (30 %, 11.1 %)
Female	13 (86.7 %, 10.3 %)∗∗	6 (85.7 %, 4.8 %)	6 (75 %, 4.8 %)	50 (48.5 %, 39.7 %)	29 (40.3 %, 23 %)	12 (80 %, 9.5 %)	28 (70 %, 22.2 %)∗∗
**Age**							
<35	5 (33.3 %, 8.9 %)	3 (42.9 %, 5.4 %)	0	28 (27.2 %, 50 %)	16 (22.2 %, 28.6 %)	5 (33.3 %, 8.9 %)	5 (12.5 %, 8.9 %)
35–44	7 (46.7 %, 10.9 %)	2 (28.6 %, 3.1 %)	2 (25 %, 3.1 %)	29 (28.2 %, 45.3 %)	24 (33.3 %, 37.5 %)	2 (13.3 %, 3.1 %)	15 (37.5 %, 23.4 %)
45–54	2 (13.3 %, 3.4 %)	2 (28.6 %, 3.4 %)	5 (62.5 %, 8.5 %)	28 (27.2 %, 47.5 %)	17 (23.6 %, 28.8 %)	3 (20 %, 5.1 %)	9 (22.5 %, 15.3 %)
55–64	1 (6.7 %, 2.6 %)	0	1 (12.5 %, 2.6 %)	12 (11.7 %, 30.8 %)	12 (16.7 %, 30.8 %)	2 (13.3 %, 5.1 %)	8 (20 %, 20.5 %)
≥65	0 (0 %, 0 %)	0	0	6 (5.8 %, 37.5 %)	3 (4.2 %, 18.8 %)	3 (20 %, 18.8 %)	3 (7.5 %, 18.8 %)
**BMI WHO Obesity Class**							
Class I	1 (6.7 %, 5.3 %)	0	0	14 (13.6 %, 73.7 %)	5 (6.9 %, 26.3 %)	2 (13.3 %, 10.5 %)	6 (15 %, 31.6 %)
Class II	4 (26.7 %, 6.6 %)	2 (28.6 %, 3.3 %)	2 (25 %, 3.3 %)	21 (20.4 %, 34.4 %)	14 (19.4 %, 23 %)	4 (26.7 %, 6.6 %)	9 (22.5 %, 14.8 %)
Class III	10 (66.7 %, 6.5 %)	5 (71.4 %, 3.3 %)	6 (75 %, 3.9 %)	68 (66 %, 44.2 %)	53 (73.6 %, 34.4 %)	9 (60 %, 5.8 %)	
**Trauma**							
Yes	10 (66.7 %, 18.9 %)	6 (85.7 %, 11.3 %)	4 (50 %, 7.55)	20 (19.4 %, 37.7 %)	16 (22.2 %, 30.2 %)	5 (33.3 %, 9.43 %)	12 (30.0 %, 22.6 %)
No	5 (33.3 %, 2.8 %)	1 (14.3 %, 0.6 %)	4 (50.0 %, 2.2 %)	83 (80.6 %, 45.9 %)	56 (77.8 %, 30.9 %)	10 (66.7 %, 5.5 %)	28 (70.0 %, 15.5 %)

Our data reveals significant associations between depression, eating disorders and abnormal eating behaviors with sex and BMI, but not with different age groups, T2DM, marriage or working status (Table 1).

In fact, female patients were more likely to be diagnosed with depression (OR = 2.4 [1.36–4.35], p-value = 0.013) and BED (OR = 6.1 [1.3–27.7], p-value = 0.036), and to have had a history of trauma at presentation (OR = 3.0 [1.5–5.9], p-value = 0.001). Furthermore, female patients were more likely to show compulsory eating behavior (OR = 2.29 [1.10–4.75], p-value = 0.007), while males engaged in hyperphagia more (OR = 2.2 [1.26,3.90], p-value = 0.001) (Table 2).

Snacking behavior was more prominent in the lower BMI Class I (73.7 %) than BMI Class II (20.4 %, OR = 5.33, p-value = 0.004) and III (44.2 %. OR = 3.54, p-value = 0.0002) (Table 2).

Those with a history of trauma were also more likely to be diagnosed with BED (OR = 8.19 [2.7–25.2], p-value = 0.0002), BN (OR = 23.0 [2.7195.5], p-value = 0.0006) (Table 2), and depression (OR = 3.76 [1.98, 7.13], p-value<0.0001), while those with depression were more likely to be diagnosed with BED (OR = 3.63 [1.24–10.62], p-value = 0.0196) or display compulsions (OR = 2.66 [1.33–5.33], p-value = 0.008) (Table 3)

**Table 3:** Association between depression and eating disorders, abnormal eating behaviors and trauma.

Count Column%	Depressed Population (N = 73)	Non-Depressed Population (N = 161)	Fisher's OR= Ratio	Fisher's P-value	Chi-Square Value	P-value
**BED**	9 (12.33 %)	6 (3.73 %)	3.63	0.0196 ∗∗	6.195	0.0128∗∗
**BN**	3 (4.11 %)	4 (2.48 %)	1.68	0.6804	0.457	0.4990
**NES**	5 (6.85 %)	3 (1.86 %)	3.872	0.1119		
**Snacking**	30 (41.10 %)	73 (45.34 %)	0.84	0.5722	0.367	0.544
**Emotional**	7 (9.59 %)	8 (4.97 %)	2.02	0.2474	1.787	0.181
**Compulsions**	20 (27.40 %)	20 (12.42 %)	2.66	0.008 ∗∗	7.948	0.0048∗∗
**Hyperphagia**	19 (26.03 %)	53 (32.92 %)	0.72	0.3592	1.12	0.2899
**Trauma**	29 (39.73 %)	24 (14.91 %)	3.76	0.000036 ∗∗	17.660	0.000036∗∗

#### 3.1.3 Conditions associations

Our multinomial logistic regression analysis with backward elimination revealed that depression was associated with unemployment (OR: 2.177 [1.183, 4.005]), trauma (OR: 3.152 [1.618, 6.139]), and compulsions (OR: 2.578 [1.233, 5.391]). Meanwhile, BED showed significant associations with trauma (OR: 9.14 [2.71, 30.77]) and the female sex (OR: 5.388 [1.113, 26.082]). Furthermore, BN was notably associated with trauma (OR: 22.94 [2.585, 203.614]). Lastly, NES was inversely related to marriage status (OR: 0.200 [0.044, 0.892])

#### 3.1.4 Subgroup analysis

We ran a subgroup analysis to determine the association between depression, eating disorders and abnormal eating behaviors among each of sex, age, BMI, marriage status, diabetic status, trauma status, working status (Table 4).

**Table 4:** Subgroup analysis with multinomial logistic regression with backward elimination between depression, eating disorders and abnormal eating behaviors, among different age groups, BMI groups, marriage, employment, diabetes and trauma status.

Demographic	Outcome	Predictor	Odds Ratio	95 % CI
**Age**				
<35	Depression	BED	13	[1.323, 127.712]
<35	BED	Depression	26.039	[2.021, 335.454]
<35	Snacking	Working	0.211	[0.051, 0.876]
35–44	Depression	Compulsions	3.165	[0.957, 10.469]
55–64	Depression	Compulsions	6.9	[1.29, 37.49]
**BMI WHO Obesity Class**				
BMI group 2	Depression	Compulsions	9.5	[1.7, 51]
BMI group 3	Depression	BED	6.5	[1.6, 26.46]
BMI group 3	BED	Depression	5.836	[1.266, 26.906]
BMI group 3	NES	Married	0.101	[0.011, 0.919]
BMI group 3	BED	Trauma	7.498	[1.670, 33.669]
BMI group 3	Bulimia	Trauma	16.25	[1.698, 155.498]
BMI group 3	NES	Trauma	6.694	[1.110, 40.362]
**Sex**				
Females	Depression	BED	2.7	[0.8, 8.8]
Females	Depression	Trauma	5.250	[2.323, 11.861]
Females	BED	Trauma	11.052	[2.809, 43.485]
Females	BED	Working	5.734	[1.289, 25.517]
Females	Bulimia	Trauma	15	[1.602, 140.465]
Males	Depression	Compulsions	4.65	[4.64, 16.17]
Males	BED	Depression	4.647	[1.335, 16.172]
**Marriage Status**				
Married	Depression	BED	5.6	[1.39, 22.63]
Married	Depression	Compulsions	3.345	[1.433, 7.808]
Married	BED	Trauma	10.671	[2.225, 51.184]
Married	Bulimia	Trauma	19.714	[1.791, 217.053]
**Trauma Status**				
No trauma	Depression	BED	13.6	[1.478, 125.155]
No trauma	Depression	Compulsions	2.836	[1.22, 6.594]
No trauma	BED	Depression	13.6	[1.478, 125.155]
Trauma	Depression	female	12.92	[1.81, 92.29]
Trauma	BED	Working	9.962	[0.730, 57.358]
**Diabetic Status**				
Not Diabetic	Depression	BED	3.231	[0.98, 10.65]
Not Diabetic	Depression	Compulsions	2.364	[1.096, 5.099]
Not Diabetic	BED	Female	11.298	[1.337, 95.486]
Not Diabetic	BED	Working	7.858	[1.409, 43.826]
Not Diabetic	BED	Trauma	9.947	[2.478, 39.921]
Not Diabetic	Bulimia	Trauma	20.571	[2.281, 185.494]
Not Diabetic	NES	Married	0.101	[0.011, 0.912]
**Working Status**				
Working	Depression	Compulsions	3.84	[1.565, 9.425]
Working	BED	Female	12	[1.373, 104.862]
Working	BED	Trauma	19.765	[4.453, 87.723]
Working	Bulimia	Trauma	14	[1.302, 150.502]

BED predicted depression in non-diabetics (OR = 3.231, 95 % CI = [0.98, 10.65]), and married individuals (OR = 5.6, 95 % CI = [1.39, 22.63]). Conversely, depression predicted BED in males (OR = 4.647, 95 % CI = [1.335, 16.172]), individuals with no trauma (OR = 13.6, 95 % CI = [1.478, 125.155]), and in younger individuals under 35 (OR = 26.039, 95 % CI = [2.021, 335.454]). The relationship is bidirectional for those with no trauma, are in BMI group 3 or aged <35, noting, respectively, for BED predicting depression: OR = 13.6, 95 % CI = [1.478, 125.155]), OR = 6.5, 95 % CI = [1.6, 26.46] and OR = 13, 95 % CI = [1.3, 127.712], and for depression predicting BED (OR = 13.6, 95 % CI = [1.478, 125.155]), OR = 5.8, 95 % CI = [1.266, 26.906] and OR = 26.039, 95 % CI = [2.021, 335.454]

Trauma predicted the development of BED or bulimia in females (OR = 11.05, 95 % CI = [2.809, 43.485] and OR = 15, 95 % CI = [1.602, 140.465]), married group (OR = 10.7, 95 % CI = [2.225, 51.184] and OR = 19.7, 95 % CI = [1.791, 217.053]), working group (OR = 19.8, 95 % CI = [4.453, 87.723] and OR = 14, 95 % CI = [1.302, 150.502]), not diabetic group (OR = 20.6, 95 % CI = [2.281, 185.494] and OR = 9.9, 95 % CI = [2.478, 39.521]), and BMI group 3 (OR = 7.5, 95 % CI = [1.678, 33.669] and OR = 16.3, 95 % CI = [1.698, 155.498]). Trauma also predicted depression in females (OR = 12.9, 95 % CI = [1.81, 92.29]) and NES in BMI group 3 (OR = 6.7, 95 % CI = [1.110, 40.362]).

Compulsive eating predicted depression in males (OR = 4.65, 95 % CI = [1.289, 25.517]), married individuals (OR = 3.345, 95 % CI = [1.433, 7.808]), working individuals (OR = 3.84, 95 % CI = [1.565, 9.425]), those without trauma (OR = 3.165, 95 % CI = [0.957, 10.469]), individuals who are not diabetic (OR = 2.364, 95 % CI = [0.596, 9.059]), those with BMI group 2 (OR = 9.5, 95 % CI = [1.7, 51]), and in the age group of 55–64 (OR = 6.9, 95 % CI = [1.29, 37.49]).

## 4 Discussion

### 4.1 Prevalence of eating disorders and depression

This study reveals the prevalence of eating disorders (12.8 %) and abnormal eating behaviors (66 %), as well as the prevalence of depression (31.2 %) and childhood trauma (22.6 %) among the participants. We were able to identify some patterns using multimodal logistical regressions, and clarify some correlations via subgroup analysis. Overall, there are positive correlations between childhood trauma, depression and BED across multiple demographics, with specificities noted among certain factors and subgroups, particularly females, or individuals who are married, are currently working, do not have diabetes, or have a BMI>40. Compulsions were also more evident in females, while hyperphagia was more seen in males.

As noted in the literature, the prevalence of eating disorders in the general population can range from 1.28 % to as high as 42.7 % [[12], [13], [14], [15], [16]].

One study in university students in France showed higher results, noting a prevalence of eating disorders of 24.8 %, with 13.3 % being bulimic eating disorders, 8.6 % hyperphagic eating disorders, and 2.9 % restrictive eating disorders [13], while another study revealed more than 50 % of young adults in France had abnormal eating behaviors, particularly binge eating disorder [14]. Our study shows lower BED and NES rates compared to the general population, noted by BED rates of 22 % in France, 27.6 % in Italy, and 42.7 % in Brazil [[17], [18], [19], [20]], and variations of NES rates between 8.9 % and 17 % [16,21,22]. Our study did not detect any differences between age groups and an eating disorder, whereas an analysis across six European countries shows younger individuals of the general population were more likely to be diagnosed with such disorders [15].

The differences of the prevalence rates and age-related differences in eating disorders within our bariatric population, contrary to findings in the general population, may be attributed to the homogenizing effect of strict surgical candidacy criteria, severity thresholds, and shared disease progression patterns specific to severe obesity, which could overshadow age-related variations typically seen in broader populations [23].

Furthermore, our study both affirms and contributes to the variability of prevalence rates seen in similar bariatric studies, ranging from 10 to 89 % [16,24,25], also explained by surgical candidacy criteria and diagnostic criteria [23].

As for certain behaviors that are not strictly classified as disorders, our study findings add to the literature, where picking and nibbling on food is reported in 30 % of patients, whereas compulsive eating is seen in 43.4 % [16,21].

The bariatric population also presents with high rates of depression. A metanalysis revealed that 19 % out of 65,363 preoperative patients were diagnosed with depression [23]. More recent studies including ours, showed slightly elevated rates up to 43 % [20,26]. Furthermore, pre-bariatric surgery patients with depression were twice as likely to have an eating disorder [19]. Our study also confirms these findings, with 2–4 times increased probability of co-diagnosing an eating disorder with depression, with even higher probabilities in subgroup analysis.

Similarly, Smith et al. reported a seven to ten times higher risk of BED diagnosis in patients reporting atypical and melancholic depressive symptoms [27]. Allison et al. and Kaur et al. found greater incidence of depressive symptoms and psychological complications in patients with BED and NES [22,28]. Ivezaj et al. observed elevated scores on depression scales among regular night eaters with a considerable effect size of 0.93, and NES has been found to be a risk factor for an earlier onset of obesity, which is related to higher rates of depression and lower self-esteem [29,30]. Mitchell et al. revealed that those with BED were more likely to be on psychiatric medications, display great depressive symptoms and possess lower self-esteem [21]. Participants who regularly indulge in loss of control (LOC)-eating and snack consumption in response to emotional states displayed higher depressive scores [31].

### 4.2 Explaining the association

The intricate relationship between eating disorders and depression manifests through various interconnected mechanisms and shared symptoms. Fabricatore et al. reported BED and depression were often precipitated by responses to negative affect, general overeating, impaired appetite regulation, and indulgence in early meals and snacks [30]. This relationship is compounded in patients with obesity who exhibited elevated levels of anxiety, depression, stress, eating behavioral disorders, and diminished self-esteem [31]. In fact, depression and low self-esteem in females contributed to BED severity noting around four times the risk of food addiction in female participants, as it was the case in our findings, noting the association between BED and depression in our female cohort [31].

The pivotal role of disinhibition as a mediator between weight bias internalization and BED was underscored among pre-surgical bariatric patients [32]. In turn, internalization and externalization of weight bias, which, respectively, denote the acceptance of negative self-perceptions related to body weight and the attribution of weight-related attitudes and stereotypes onto others, have been associated with a heightened sense of loss of control during eating episodes [33]. Dahl et al. further illuminated this nexus by demonstrating that patients with eating disorders, inclusive of subthreshold BED, exhibited markedly higher depressive and neuroticism scores, irrespective of sex and age demographics, suggesting a pervasive influence of depressive and neurotic symptoms across the disordered eating spectrum [34]. However, this association is yet incompletely explained, as it is possible that patients with BED reported higher disinhibition and hunger, despite having similar depression levels to those without an eating disorder and low self-esteem levels [35].

### 4.3 The impact of prior childhood trauma

An attempt to mediate this relationship revealed a traumatic childhood is likely to influence the development of eating disorders and depression, possibly via alterations in the hypothalamic-pituitary-adrenal axis [36,37]. Orcutt et al. showed that childhood neglect and physical, emotional or mental abuse increase the risk of BED, depression, and suicidal ideations, even after account for other factors such as age, education or BMI [38].

The association between trauma and eating disorders could potentially stem from the inception of dysfunctional core beliefs, specifically those categorized within the disconnection and rejection domain. This involves the inability to form secure attachments and challenges in interpersonal relationships [39]. Similarly, low resilience was found in patients with both depression and childhood trauma in bariatric patients, and in turn, depression seems to be a unique independent mediator between adverse childhood experiences and obesity [40].

### 4.4 Strengths and limitations

Our study's primary strength lies in its comprehensive integration of multiple mental health dimensions previously studied separately in the bariatric population, including depression, childhood trauma, eating disorders, and abnormal eating behaviors, along with their complex interconnections. This integrated approach, supported by interviews conducted by specialized psychologists and psychiatrists using ICD-10 criteria, provides a more nuanced understanding of the psychological profile of bariatric surgery candidates. However, it is essential to acknowledge the study's limitations, as it was conducted at a single public hospital center where patients undergo a lengthy preoperative course that requires resolution of depression and eating disorders before surgical clearance. This requirement may have led some patients to withhold crucial information to expedite their clearance process. Despite these limitations, our findings emphasize the significant role of childhood trauma in various psychiatric presentations and eating disorders, as well as the impact of social factors such as employment and marital status on mental health outcomes. The study underscores the importance of comprehensive preoperative psychological evaluation and treatment with appropriate medications or behavioral therapies to facilitate a smooth pre-operative course and limit post-operative weight gain rebound.

Future research should focus on longitudinal post-surgical outcomes in patients with pre-existing mental health conditions, the development and validation of comprehensive screening tools that account for co-occurring conditions, and the evaluation of targeted interventions that address both trauma history and gender-specific vulnerabilities in the bariatric population.

## 5 Conclusion

The high prevalence and association between depression and eating disorders among pre-bariatric surgery patients emphasize the crucial need for comprehensive mental health assessments before the surgery. Our study, with a larger sample size and relatively balanced population compared to similar research, underscores the significance of screening specific demographics for depressive and eating disorders, and, for certain eating behaviors and presence of trauma. Integrating these evaluations into every preoperative process is essential to identify relapse and deterioration following the bariatric surgery. Moreover, recognizing the significance of sociodemographic factors and their connection to depression and certain eating disorders can guide our research efforts. A thorough understanding of these factors can contribute to effective preoperative preparation and risk assessment, which are key factors for achieving successful outcomes in bariatric surgery.

## CRediT authorship contribution statement

**Andrew El Alam:** Writing – review & editing, Writing – original draft, Visualization, Validation, Software, Project administration, Methodology, Investigation, Formal analysis, Data curation, Conceptualization. **Mohamad Fleifel:** Writing – review & editing, Writing – original draft, Visualization, Validation, Methodology, Investigation, Formal analysis, Data curation, Conceptualization. **Hicham Baba:** Writing – review & editing, Writing – original draft, Visualization, Software, Resources, Methodology, Investigation, Formal analysis, Conceptualization. **Souha Bayda:** Writing – original draft, Visualization, Validation, Software, Methodology, Formal analysis, Conceptualization. **Bertha Maria Nassani:** Writing – original draft, Visualization, Validation, Project administration, Methodology, Investigation, Conceptualization. **Jocelyne Azar:** Writing – original draft, Visualization, Validation, Supervision, Methodology, Investigation, Formal analysis. **Arnaud Monier:** Writing – original draft, Visualization, Validation, Supervision, Conceptualization.

## Declaration of competing interest

The authors declare that they have no known competing financial interests or personal relationships that could have appeared to influence the work reported in this paper.
